# Gender Differences in the Association Between Marriage and Cognitive Decline: Evidence From the China Health and Retirement Longitudinal Study (CHARLS)

**DOI:** 10.1002/pchj.70119

**Published:** 2026-08-08

**Authors:** Yanfei Zhang, Jin Wang, Tianqiang Zhang, Guorui Liu

**Affiliations:** ^1^ Department of Medical Psychology Second Affiliated Hospital of Naval Medical University Shanghai China; ^2^ Department of Medical Psychology The 908th Hospital of Joint Logistic Support Force Nanchang China; ^3^ Central Sterile Supply Department Third Affiliated Hospital of Naval Medical University Shanghai China; ^4^ Department of Medical Psychology No. 905 Hospital of PLA Navy Shanghai China

**Keywords:** aging, CHARLS, cognitive decline, gender differences, longitudinal study, marital status

## Abstract

This study aimed to investigate the relationship between marital status and the risk of cognitive decline in middle‐aged and older adults, with a particular focus on gender differences. Data were derived from the China Health and Retirement Longitudinal Study (CHARLS), using Cox proportional hazards models to examine the association between marital status and cognitive decline. The analysis controlled for sociodemographic factors (age, education, smoking, alcohol consumption), lifestyle factors (physical activity, sleep), and gender. Gender‐stratified analyses were conducted to explore potential differences in the protective effects of marriage on cognitive function. The overall association between marital status and cognitive decline risk did not reach statistical significance after controlling for confounders. However, gender‐stratified analysis revealed that marriage was significantly negatively associated with cognitive decline risk in men. No significant association was observed in women. Age remained a significant predictor of cognitive impairment. Marriage may be associated with cognitive function, particularly among men, while no significant association was observed in women. These findings suggest that gender differences should be taken into account in cognitive health interventions. Future studies are warranted to explore the potential mediating roles of marital quality, social support, and emotional interactions in shaping cognitive outcomes.

## 1 Introduction

With aging, individuals generally experience a decline in cognitive function. This decline not only affects the quality of life in older adults but may also serve as an early sign of dementia and other neurocognitive disorders (Chen et al. 2024; Haghighi et al. 2024). The decline in cognitive abilities has widespread and diverse negative impacts on both psychological and physical health, and it places a significant burden on families and healthcare systems (Zhi et al. 2025). Therefore, preventing or delaying cognitive decline is of paramount importance. In recent years, the influence of social and psychological factors on cognitive health has gained increasing attention, with maintaining strong social relationships being considered a potential cognitive protective factor (Livingston et al. 2020; Wang et al. 2022). In adulthood, marital relationships are particularly important for the benefits individuals derive from their social connections. Studies have shown that married individuals typically have higher levels of social support, more stable life structures, and more regular lifestyles, all of which may collectively contribute to the preservation of cognitive function (Jennings et al. 2022).

Some studies have shown that married individuals exhibit better cognitive performance compared to unmarried, divorced, or widowed individuals (Jadenur et al. 2022; Zhang et al. 2024). However, findings on the influence of marital status on cognitive function across different genders are not entirely consistent (Sundström et al. 2016). Research has indicated that in Korea, women are more likely than men to experience cognitive impairment following widowhood (Park et al. 2023). Other studies have found that among single individuals, men have a higher risk of developing dementia than women (Feng et al. 2014; Liu et al. 2020). Studies on gender differences have shown that in developing countries, women are more prone to cognitive issues than men, whereas the opposite is true in developed countries (Gazbare and Palekar 2024). Furthermore, other research suggests that the impact of marital status on cognitive outcomes does not differ by gender (Liu et al. 2019). Men and women differ systematically in terms of social roles, health behaviors, and social support networks, and these differences may lead to divergent patterns of how marriage affects cognitive function across genders (Jadenur et al. 2022).

Although gender differences in cognitive health have gradually gained attention, gender‐specific evidence on the protective effects of marriage on cognitive function remains relatively limited, especially in large‐scale longitudinal studies of elderly populations in China. A deeper exploration of gender differences in the relationship between marital status and cognitive function is crucial for understanding the mechanisms of social relationships and cognitive aging, as well as for formulating targeted cognitive health promotion strategies and social support policies.

Therefore, this study utilizes large‐scale longitudinal data from the China Health and Retirement Longitudinal Study (CHARLS) to systematically analyze the association between marital status and cognitive function, with a particular focus on the moderating role of gender. By constructing multilevel regression models and conducting gender‐stratified analysis, this study aims to reveal the gender heterogeneity in the protective effects of marriage on cognitive function, providing empirical evidence for cognitive health interventions targeting the elderly population in China.

## 2 Methods

### 2.1 Study Population

The data for this study were derived from the China Health and Retirement Longitudinal Study (CHARLS), jointly conducted by Wuhan University and Peking University, which is publicly available (http://charls.pku.edu.cn/). This study included data from the baseline survey conducted in 2011 and the fifth‐round survey in 2020. CHARLS employs a multi‐stage stratified sampling design, covering 28 provinces in China (excluding Tibet, Taiwan, Hong Kong, and Macau), with a total of 150 counties and 450 communities. It systematically collects demographic characteristics, health status, physical activity, and socioeconomic information from respondents. Strict quality control measures are implemented at each stage of the survey to ensure the reliability and validity of the data. For this analysis, the initial sample size was 17,597 participants. After matching participant IDs with the 2020 follow‐up dataset, 13,659 participants remained. Among them, 1028 participants were excluded due to missing data, and 12,631 participants with complete data on all variables were included in the final Cox regression analysis.

### 2.2 Ethical Statement

CHARLS received approval from the Biomedical Ethics Review Committee of Peking University in 2008 (IRB00001052‐11015). All participants signed written informed consent forms prior to participation in the survey. The methods employed in this study comply with the applicable protocols and guidelines of CHARLS. All participants voluntarily joined CHARLS after signing the informed consent form.

### 2.3 Measurements

#### 2.3.1 Participant Characteristics

Basic information on participants was extracted from the questionnaire, including age, gender, education level, and marital status. Education level was classified into two groups: low (including no schooling, primary, junior high, and high school) and high (3‐Year College/Associate degree or above). Marital status was treated as a binary variable, with married individuals coded as 1 and non‐married individuals (including divorced, widowed, separated, or never married) coded as 0.

#### 2.3.2 Physical Activity (PA) Assessment

The intensity and duration of physical activity (PA) were derived from items DA051, DA052, DA053, DA054, and DA055 of the CHARLS questionnaire. Respondents reported whether they engaged in the following activities during the past week: Vigorous physical activity (VPA): such as heavy lifting, digging, farming, fast cycling, or cycling with loads; Moderate physical activity (MPA): such as light lifting, cycling at a normal speed, cleaning, Tai Chi, or brisk walking; Light physical activity (LPA): primarily walking, including walking for work, household chores, leisure, or exercise. Respondents could report multiple activities of varying intensities. The frequency of exercise was also recorded by asking how many days per week the respondent engaged in physical activity.

The duration of each activity was assessed using a tiered questioning approach: First, respondents were asked whether they engaged in physical activity for < 2 h (coded as 1) or ≥ 2 h (coded as 2) per session. If the response was < 2 h, a follow‐up question asked whether the activity lasted < 30 min (coded as 1) or ≥ 30 min (coded as 2). If the response was ≥ 2 h, further differentiation was made between < 4 h (coded as 1) or ≥ 4 h (coded as 2). The responses were then converted into an approximate daily duration in hours: < 30 min = 0.25 h/day; 30 min to 2 h = 1 h/day; 2 to 4 h = 3 h/day; ≥ 4 h = 5 h/day.

#### 2.3.3 Metabolic Equivalents (METs)

Based on the duration and frequency of activities, moderate‐to‐vigorous physical activity (MVPA) was converted into metabolic equivalents (METs) using the following formulas: Moderate intensity: 4.0 × duration per session × frequency; Vigorous intensity: 8.0 × duration per session × frequency. Finally, individuals were categorized into low and high MET groups based on their MET levels.

#### 2.3.4 Cognitive Function

Cognitive decline was determined based on two variables from CHARLS: EXDA010 and DA002. EXDA010: The question asks, “At what age did you first notice significant memory problems?” This is used to infer the onset of cognitive decline. DA002: This question asks about changes in memory‐related diseases (such as dementia, brain atrophy, etc.) compared to the last follow‐up. Responses include “better,” “about the same,” “worse,” or “did not occur at last follow‐up.” Responses of “better,” “about the same,” or “worse” were considered indicative of cognitive decline at the last follow‐up. A response of “did not occur at last follow‐up” was considered indicative of new cognitive decline at the current follow‐up.

In the Cox proportional hazards model, the event is defined as the first occurrence of cognitive decline. Censoring occurs due to death, loss to follow‐up, or the end of the 2020 survey, with the earliest event determining the censoring time.

#### 2.3.5 Follow‐Up Time

Follow‐up time is defined as the time from the baseline survey date to the date of death, loss to follow‐up, or the end of the follow‐up period, calculated in years.

### 2.4 Covariates

Potential confounding factors include age, gender, smoking status, alcohol consumption, and depression symptoms. Smoking and alcohol consumption were based on self‐reported current status. Specifically, for smoking, “Yes (1)” indicates individuals who have ever chewed tobacco, smoked a pipe, smoked self‐rolled cigarettes, or smoked cigarettes/cigars, specifically referring to those who have smoked more than 100 cigarettes in their lifetime. “No (2)” indicates non‐smokers. For alcohol consumption, “More than once a month (1)” indicates frequent drinkers, “Less than once a month (2)” indicates occasional drinkers, and “No (3)” indicates individuals who do not drink. Depressive symptoms were assessed using the CES‐D 10, a 10‐item scale with 4‐point responses (0–3), where higher scores indicate more severe symptoms.

Sleep Duration: The sleep duration variable was derived from the CHARLS item DA049, which asks, “On average, how many hours do you sleep each night over the past month?”. Sleep duration was categorized into three groups based on commonly used thresholds in previous studies: short sleep (< 6 h), normal sleep (6–8 h), and long sleep (> 8 h). For statistical analysis, these categories were coded as 1 (< 6 h), 2 (6–8 h), and 3 (> 8 h).

### 2.5 Statistical Analysis

For handling missing data, we employed listwise deletion. This method ensures that only complete cases with no missing values for the variables of interest were included in the analysis. To explore the association between marital status and the risk of cognitive decline, as well as the gender differences in this association, this study used stepwise Cox proportional hazards models for analysis. Model 1 (Basic model): Includes age, gender, marital status, education level, and the “gender × marital status” interaction term. Model 2 (Extended model): Further adjusts for lifestyle factors, including smoking, alcohol use, sleep duration, depressive symptoms, and physical activity (MVPA categories) in addition to the variables in Model 1. Model 3 (Subgroup analysis): Stratifies by gender and performs separate Cox regressions to test whether the effect of marital status differs between genders. Categorical variables are dummy‐coded as necessary. Results are presented as hazard ratios (HR) with 95% confidence intervals (CI). To assess whether the Cox models met the proportional hazards assumption, we conducted the Schoenfeld residuals test. The results indicated that the proportional hazards assumption was not violated for any of the variables, suggesting that the models meet the assumption. All statistical analyses were performed using SPSS 26.0, with a significance level set at a two‐sided *α* = 0.05. Sex‐stratified analyses were conducted to explore potential differences between males and females.

## 3 Results

### 3.1 Sample Characteristics

As shown in Table 1, a total of 13,659 participants were included in this study, of whom 6379 were male (46.7%) and 7280 were female (53.3%). The minimum age was 45 years. In terms of educational attainment, 45.0% had low education levels (no formal education or below primary school), 43.2% had moderate education (primary or junior high school), and 11.8% had high school or higher education. The majority were married (82.0%), while 18.0% were single (including divorced, widowed, never married, or separated) at baseline. The average follow‐up time was 8.97 years, during which 106 cases of cognitive decline were observed.

**TABLE 1:** Baseline characteristics of participants by gender and marital status.

Characteristic	Total (*n*)	Male (*n*)	Female (*n*)	Married (*n*)	Non‐married (*n*)
Age, mean ± SD	66.87 ± 9.20 (*n* = 13,624)	67.33 ± 8.87 (*n* = 6374)	66.46 ± 9.47 (*n* = 7250)	66.19 ± 8.64 (*n* = 11,188)	69.99 ± 10.89 (*n* = 2446)
Sleep duration, *n* (%)					
Sleep duration (1)	3620 (28.7%)	1485 (25.7%)	2134 (31.2%)	2928 (27,4%)	692 (35.5%)
Sleep duration (2)	7959 (63.0%)	3814 (66.0%)	4141 (60.5%)	6881 (64.4%)	1078 (55.3%)
Sleep duration (3)	1051 (8.3%)	477 (8.3%)	571 (8.3%)	872 (8.2%)	179 (9.2%)
Education level, *n* (%)					
Low (no schooling, primary, junior high, high school)	6139 (45.0%)	1908 (29.9%)	4231 (58.2%)	4746 (42.3%)	1393 (57.1%)
High (3‐Year College/Associate degree and above)	1609 (11.8%)	1032 (16.2%)	577 (7.9%)	1438 (12.8%)	171 (7.0%)
Marital status, *n* (%)					
Married	11,203 (82.0%)	5384 (84.4%)	5819 (80.0%)	—	—
Non‐married	2445 (18.0%)	995 (15.6%)	1450 (20.0%)	—	—
Smoking, *n* (%)					
Yes (1)	5239 (38.4%)	4700 (73.6%)	539 (7.4%)	4354 (38.9%)	887 (36.4%)
No (2)	8394 (61.6%)	1672 (26.2%)	6722 (92.6%)	6846 (61.1%)	1556 (63.6%)
Drinking, *n* (%)					
More than once a month (1)	3436 (25.2%)	2929 (45.9%)	507 (7.0%)	2883 (25.7%)	555 (22.8%)
Less than once a month (2)	1124 (8.2%)	743 (11.6%)	381 (5.2%)	931 (8.3%)	194 (7.9%)
No (3)	9068 (66.6%)	2695 (42.3%)	6373 (87.7%)	7386 (65.9%)	1689 (69.3%)
Physical activity level, *n* (%)					
Low (1)	8995 (65.9%)	4309 (67.5%)	4686 (64.5%)	7253 (64.7%)	1730 (73.1%)
High (2)	4654 (34.1%)	2070 (32.5%)	2584 (35.5%)	3957 (35.3%)	637 (26.9%)

### 3.2 Association Between Marital Status and the Risk of Cognitive Decline

#### 3.2.1 Model 1: Basic Model

The final sample size included in the Cox regression analysis was *n* = 12,631. As shown in Table 2, after adjusting for age, education level, and gender, the association between marital status and the risk of cognitive decline was not statistically significant (HR = 0.721, 95% CI: 0.392–1.328, *p* = 0.294). Age was significantly positively correlated with the risk of cognitive decline (HR = 1.121, 95% CI: 0.902–2.544, *p* < 0.001), indicating that the risk of cognitive decline increases significantly with age. There was a negative trend between education level and cognitive decline risk, with higher education levels associated with a lower risk of cognitive decline, though this relationship was not significant (low vs. high: HR = 0.772, *p* = 0.719). The gender effect showed that the risk of cognitive decline was slightly lower in women compared to men (HR = 1.515, 95% CI: 0.902–2.544, *p* = 0.116). The interaction term between gender and marital status was not significant (*p* = 0.387).

**TABLE 2:** Cox proportional hazards model 1 (basic model).

Variable	*B*	SE	Wald	df	*p*	HR (Exp(*B*))	95% CI Lower	95% CI Upper	Notes
Age	0.112	0.01	137.729	1	< 0.001	1.121	1.1	1.142	Continuous
Sex (Male = 1)	0.415	0.264	2.469	1	0.116	1.515	0.902	2.544	Categorical
Education (Low = 1)	−0.259	0.719	0.130	1	0.719	0.772	0.189	3.159	Categorical
Marital status (Married = 1)	−0.327	0.312	1.100	1	0.294	0.721	0.392	1.328	Categorical
Sex × Marital status	−0.36	0.416	0.749	1	0.387	0.697	0.308	1.577	Interaction

#### 3.2.2 Model 2: Extended Model

As shown in Table 3, after further adjusting for lifestyle factors such as smoking, alcohol consumption, physical activity, and sleep duration, the association between marital status and the risk of cognitive decline remained largely unchanged, with no significant variation observed (HR = 0.618, *p* > 0.05). Age remained a significant risk factor, and higher education level continued to show a potential protective trend. Among lifestyle variables, regular physical activity and longer sleep duration were both positively associated with better cognitive function, although these differences were not statistically significant (*p* > 0.05).

**TABLE 3:** Cox proportional hazards model 2 (extended model).

Variable	*B*	SE	Wald	df	*p*	HR (Exp(*B*))	95% CI Lower	95% CI Upper
Age	0.118	0.011	120.873	1	< 0.001	1.127	1.103	1.151
Sex (Male = 1)	0.031	0.339	0.008	1	0.928	1.031	0.530	2.005
Education (Low = 1)	−0.424	0.723	0.344	1	0.557	0.654	0.159	2.700
Marital status (Married = 1)	−0.482	0.33	2.135	1	0.144	0.618	0.324	1.179
Sex × Marital status	−0.201	0.44	0.208	1	0.648	0.818	0.345	1.940
Physical activity (Low = 1)	0.118	0.225	0.273	1	0.601	1.125	0.724	1.749
Sleep duration (1)	−0.213	0.371	0.330	1	0.566	0.808	0.390	1.673
Sleep duration (2)	−0.081	0.349	0.063	1	0.818	0.923	0.465	1.830
Smoking (1)	0.413	0.264	2.446	1	0.118	1.511	0.901	2.533
Drinking (1)	0.298	0.254	1.377	1	0.241	1.347	0.819	2.214
Drinking (2)	0.132	0.439	0.091	1	0.763	1.141	0.483	2.696
Depressive symptoms	0.012	0.020	0.348	1	0.555	1.012	0.973	1.052

#### 3.2.3 Model 3: Subgroup Analysis (Gender Stratification)

In sex‐stratified analyses, marital status appeared to be associated with cognitive impairment among males but not among females, although these findings should be interpreted with caution. To further explore potential gender‐specific pathways, separate Cox proportional hazards models were conducted for men and women. As shown in Table 4, marital status was not significantly associated with the risk of cognitive decline among females (HR = 0.500, 95% CI: 0.259–0.966, *p* > 0.05). In contrast, among males, being married was associated with a lower risk of cognitive decline (HR = 0.489, 95% CI: 0.253–0.945, *p* < 0.05) (Table 5). In sensitivity analyses using a four‐category marital status variable, married, divorced/separated, and widowed individuals had a lower risk of cognitive impairment compared with those who had never married (Table S1).

**TABLE 4:** Cox model 3: Sex‐stratified analysis female.

Variable	*B*	SE	Wald	df	*p*	HR	95% CI Lower	95% CI Upper
Age	0.154	0.017	80.951	1	< 0.001	1.166	1.128	1.206
Education (Low = 1)	0.595	0.633	0.883	1	0.347	1.813	0.524	6.267
Marital status (Married = 1)	−0.693	0.336	4.256	1	0.039	0.500	0.259	0.966
Physical activity (Low = 1)	−0.257	0.317	0.658	1	0.417	0.773	0.415	1.439
Sleep duration (1)	−0.128	0.563	0.052	1	0.820	0.880	0.292	2.655
Sleep duration (2)	−0.171	0.548	0.098	1	0.755	0.843	0.288	2.466
Smoking	0.718	0.349	4.234	1	0.051	2.051	1.035	4.064
Drinking (1)	0.092	0.480	0.037	1	0.848	1.096	0.428	2.810
Drinking (2)	0.291	0.728	0.160	1	0.690	1.338	0.321	5.574
Depressive symptoms	0.007	0.027	0.066	1	0.798	1.007	0.955	1.062

**TABLE 5:** Cox model 3: Sex‐stratified analysis male.

Variable	*B*	SE	Wald	df	*p*	HR	95% CI Lower	95% CI Upper
Age	0.088	0.015	35.647	1	< 0.001	1.092	1.061	1.123
Education (Low = 1)	−0.627	0.729	0.739	1	0.390	0.534	0.128	2.230
Marital status (Married = 1)	−0.716	0.336	4.532	1	0.033	0.489	0.253	0.945
Physical activity (Low = 1)	0.397	0.325	1.491	1	0.222	1.488	0.786	2.816
Sleep duration (1)	−0.466	0.513	0.826	1	0.363	0.627	0.230	1.715
Sleep duration (2)	−0.134	0.454	0.087	1	0.769	0.875	0.359	2.131
Smoking	−0.004	0.331	1.769	1	0.977	0.990	0.520	1.906
Drinking (1)	0.358	0.308	1.349	1	0.245	1.430	0.782	2.617
Drinking (2)	0.057	0.552	0.011	1	0.918	1.059	0.359	3.126
Depressive symptoms	0.021	0.029	0.497	1	0.481	1.021	0.964	1.081

### 3.3 Model Robustness and Consistency Tests

Across the different models (Models 1–3), the direction and significance of the main covariates (age, education, marital status) remained consistent, suggesting that the model results are robust. After Bonferroni correction, age remained the most significant risk factor for cognitive decline (adjusted *p* < 0.001).

Moreover, in the gender‐stratified analysis, the direction of the association between marital status and cognitive decline did not reverse, further supporting the robustness of the gender differences.

## 4 Discussion

This study, based on longitudinal data from the China Health and Retirement Longitudinal Study (CHARLS) 2011 and 2020, employed Cox proportional hazards models to investigate the relationship between marital status and the risk of cognitive decline in middle‐aged and older adults, and further analyzed gender differences. The results showed that after controlling for sociodemographic factors such as age and education level, as well as lifestyle variables, the overall association between marital status and the risk of cognitive decline did not reach statistical significance. However, gender‐stratified analysis indicated that marriage was associated with a lower risk of cognitive decline in the male group, with no significant association found in the female group.

This finding is consistent with some previous studies. Several longitudinal studies have found that marriage or having a partner can reduce the risk of cognitive decline and dementia (Sommerlad et al. 2018). Marriage is believed to promote cognitive health through various mechanisms, including social support, emotional comfort, reinforcement of healthy behaviors, and the sharing of economic resources (Haghighi et al. 2024; Perry et al. 2022). However, our study suggests that the association between marriage and cognitive function may vary by gender. The risk of cognitive decline associated with marital experiences in men and women is likely shaped by specific social contexts, including differing family and gender roles. Men appear to experience more notable associations with marriage, which may be related to their greater reliance on partners for social support and emotional regulation within the marriage (Shen et al. 2022). In contrast, women have more diverse social connections and, even in widowhood or single status, can maintain social interactions and psychological health through friends, family, or community networks (Babaei et al. 2024; Shen et al. 2022).

The results regarding gender differences carry important social psychological implications. For men, marriage may delay cognitive decline by providing stable emotional support, promoting healthy behaviors (such as regular medical visits and reducing harmful habits), and other similar mechanisms (Feng et al. 2014). In contrast, women may bear more caregiving and family‐related pressures within marriage, which could counteract some of the health benefits marriage provides (Liu et al. 2020). This “gender‐asymmetrical effect of marriage” is especially pronounced in the Chinese sociocultural context, where traditional gender roles and caregiving burdens may prevent women from receiving the same cognitive protection benefits from marriage (Shen et al. 2022).

After adjusting for factors such as education, age, smoking, alcohol use, physical activity, and sleep, the association between marital status and overall cognitive risk diminished, indicating that some of the observed relationships may be attributed to variations in sociodemographic and lifestyle factors (Wang et al. 2023). Education level consistently appeared as an important protective factor in the models, consistent with previous studies, indicating that higher education may reduce the risk of cognitive decline by enhancing cognitive reserve (Jadenur et al. 2022). Additionally, age was a significant predictor of cognitive decline, further supporting the stable relationship between aging and cognitive impairment (Lv et al. 2024).

The strength of this study lies in the use of large‐scale, nationally representative longitudinal data, with Cox survival analysis effectively capturing the time dimension of cognitive changes. However, this study has several limitations. First, although a longitudinal design was employed, causal inference should be made with caution due to difficulties in extracting and analyzing certain variables from the database. Several potential confounding factors, such as marital quality, levels of social support, and chronic disease burden, were not fully incorporated into the analysis. Second, marital status only reflects the current marital situation. Although CHARLS categorizes marital status into five groups—unmarried, married but separated, cohabiting, divorced, and widowed—the small sample size of some groups (e.g., divorced or long‐term single) prevented the differentiation of specific subtypes such as widowed, divorced, or long‐term single, which may obscure the differential effects of various types of non‐marital status on cognitive function.

## 5 Conclusion

In conclusion, although sex‐stratified analyses were conducted to explore potential gender differences, the interaction between sex and marital status was not statistically significant. As multiple subgroup analyses can increase the risk of type I error, these findings should be interpreted with caution. Future studies could further investigate the mediating mechanisms of marital quality, social support networks, and emotional interactions, and explore the complex relationship between marriage and cognitive health from a gendered perspective. This would provide a basis for developing gender‐sensitive strategies for the prevention of cognitive decline.

## Funding

The authors have nothing to report.

## Ethics Statement

The CHARLS study was first approved by the Ethics Committee of Peking University in 2008 (IRB00001052‐11,015). All procedures in this study adhered to the applicable CHARLS guidelines and regulations.

## Consent

All participants provided informed consent before voluntarily enrolling in CHARLS.

## Conflicts of Interest

The authors declare no conflicts of interest.

## Supporting information


**Table S1:** Sensitivity analysis of marital status categories and risk of cognitive impairment.

## Data Availability

The study uses secondary data which is available on reasonable request through http://charls.pku.edu.cn/.
