# Supplementary material for: Gender Differences in the Association Between Marriage and Cognitive Decline: Evidence From the China Health and Retirement Longitudinal Study (CHARLS)
Source: Psych J. 2026 Aug 8;15(4):e70119. doi: 10.1002/pchj.70119 (PMC13451936; doi:10.1002/pchj.70119)
Supplement: Supplementary file 1 [file PCHJ-15-e70119-s001.docx]

| **Supplementary Table S1. Sensitivity analysis of marital status categories and risk of cognitive impairment** | | | |
| --- | --- | --- | --- |
| **Variable** | **HR** | **95% CI** | ***P*** |
| **Marital status** |  |  |  |
| Never married | Reference |  |  |
| Married | 0.200 | 0.071 – 0.562 | 0.002 |
| Divorced / Separated | 0.208 | 0.055 – 0.790 | 0.021 |
| Widowed | 0.300 | 0.100 – 0.902 | 0.032 |
| **Covariates** |  |  |  |
| Age (per year) | 1.124 | 1.101 – 1.148 | < 0.001 |
| Sex (Male vs Female) | 1.014 | 0.591 – 1.738 | 0.961 |
| Smoking (Current vs Non-smoker) | 1.474 | 0.898 – 2.420 | 0.125 |
| Drinking (> 1/month vs None) | 1.154 | 0.711 – 1.873 | 0.561 |
| Drinking (< 1/month vs None) | 1.078 | 0.507 – 2.289 | 0.846 |
| Physical activity (Medium vs Low) | 1.190 | 0.771 – 1.836 | 0.432 |
| Education (Medium vs Low) | 0.707 | 0.172 – 2.901 | 0.630 |
| Notes: HR = hazard ratio; CI = confidence interval. Cox proportional hazards models were adjusted for age, sex, smoking status, alcohol consumption, physical activity level, and education level. Marital status was categorized into four groups: married, divorced/separated, widowed, and never married. Never married participants were used as the reference group. | | | |
